# Short-term results of the efficacy of percutaneous tibial nerve stimulation on urinary symptoms and its financial cost

**DOI:** 10.4274/jtgga.2017.0115

**Published:** 2018-03-01

**Authors:** Zehra Kurdoğlu, Danielle Carr, Jihad Harmouche, Serdar Ünlü, Gökhan S. Kılıç

**Affiliations:** 1 Department of Obstetrics and Gynecology, Ankara Training and Research Hospital, Ankara, Turkey; 2 Department of Obstetrics and Gynecology, The University of Texas Medical Branch, Texas, USA

**Keywords:** Transcutaneous electric nerve stimulation, tibial nerve, urinary bladder, overactive, costs and cost analysis, lower urinary tract symptoms, office visits

## Abstract

**Objective:**

Overactive bladder (OAB) affects 16.9% of women in the United States. Percutaneous tibial nerve stimulation (PTNS) is a third-line treatment for patients who are refractory to behavioral and pharmacologic therapies. We aimed to evaluate the effects of PTNS on urinary symptoms in patients diagnosed as having refractory OAB and investigate the cost of medications and clinical visits before and after PTNS treatment.

**Material and Methods:**

We reviewed 60 women with refractory OAB treated with PTNS. Episodes of urinary frequency, leakage, urgency, and nocturia; number of follow-up visits; and medications were recorded. The mean quarterly drug, physician, nurse, and provider costs were calculated. The episodes of urinary symptoms, numbers of follow-up visits, and costs of medications and visits before and after PTNS were compared.

**Results:**

Of the 60 patients with refractory OAB, 24 patients who completed 12 weekly sessions of initial PTNS were evaluated. The number of urinary symptoms and follow-up visits significantly decreased after PTNS (p<0.05). The average quarterly medication cost decreased from $656.36±292.45 to $375.51±331.79 after PTNS (p=0.001). After PTNS, quarterly physician and nurse visit costs decreased from $81.73±70.39 to $25.89±54.40 and from $55.23±38.32 to $15.53±19.58, respectively (p<0.05). The quarterly total provider cost was similar before and after PTNS.

**Conclusion:**

PTNS treatment significantly improved urinary symptoms of patients with refractory OAB and reduced the costs of medications and physician and nurse visits.

## Introduction

Overactive bladder (OAB) syndrome is defined as "urgency, with or without urge incontinence, usually with frequency and nocturia in the absence of proven infection or other obvious pathology" by the International Continence Society (1). The prevalence of OAB varies between 12.8% and 17.4% and increases with age for both sexes (2,3). In the United States (US), its prevalence is 16.0% in men and 16.9% in women, and 29.8 million adults aged ≥40 years are estimated to have OAB symptoms (4,5).

The American Urological Association and the Society of Urodynamics, Female Pelvic Medicine and Urogenital Reconstruction suggest that the first-line treatment should be composed of diet and behavioral therapies such as reducing caffeine, alcohol, and fluid intake; weight loss; pelvic floor physical therapy; timed voiding; and bladder training. Antimuscarinic therapies may be added to behavioral therapies at the same time. Second-line treatment should include pharmacologic agents, antimuscarinics or oral β3-adrenoceptor agonists for a minimum of 3 months. Adverse effects of medication or inadequate symptom relief may lead to discontinuation of pharmacologic therapy. Intradetrusor botulinum toxin A, percutaneous tibial nerve stimulation (PTNS), and sacral nerve stimulation (SNS) may be recommended as third-line therapies to patients who are refractory to behavioral and pharmacologic therapies (6).

PTNS is the least invasive form of neuromodulation, which uses electrical stimulation to afferent fibers of the posterior tibial nerve (L4-S3) through a needle electrode inserted 3-5 cm cephalad to the medial malleolus. The needle is connected to a device that provides 0.5-9 mA electrical stimulation at 20 Hz. A grounding pad is placed on the bottom of the foot. Nerve stimulation is confirmed by flexion of the big toe and sensory stimulation on the bottom of the foot is created. The exact mechanism of PTNS is unknown. However, it seems that afferent nerve stimulation leads to activation of inhibitory sympathetic neurons through a direct sacral route and modulates the efferent outflow to the lower urinary tract. Treatment is composed of 12 weekly stimulation sessions lasting 30 minutes each. After this initial treatment, sessions may be tapered or maintained according to symptom improvement (7).

The aim of the present study was to evaluate the effects of PTNS on urinary symptoms in patients diagnosed as having refractory OAB and to analyze the cost of medications and clinical visits before and after PTNS treatment.

## Material and Methods

In this retrospective study, we reviewed patients who were diagnosed as having refractory OAB and treated with PTNS in the Urogynecology Clinic of the University of Texas Medical Branch, a tertiary-care center in Galveston, Texas. Sixty patients who were diagnosed as having OAB and who were resistant to treatment with anticholinergic medications and behavioral modification participated in this study. Forty-six of the 60 patients had undergone at least 1 PTNS session. Twenty-four had completed 12 weekly PTNS sessions. The weekly stimulation sessions lasted 30 minutes and were performed in an outpatient setting. We included women who were aged 21-85 years, who were diagnosed with refractory OAB, and who had completed 12 weekly PTNS sessions. Pregnant women, those with cancer, pacemaker users, Texas Department of Criminal Justice inmates, women who had urinary retention or urinary tract obstruction, and those whose symptoms were suspected to be neurologic or inflammatory in origin were excluded. The study was approved by the Institutional Review Board (IRB: 16-0219).

First, all patients undergoing PTNS treatment were identified using the current procedural terminology code 64566, which is a medical procedural code under the category - neurostimulator procedures on peripheral nerves. As maintained by the American Medical Association, the procedure was billed under this code, with the descriptor "posterior tibial neurostimulation, percutaneous needle electrode, single treatment, including programming". Then age; body mass index (BMI); episodes of urinary frequency, urgency, nocturia, and incontinence; and numbers of physician and nurse visits were obtained from electronic medical records. Medications used and clinic visits were reviewed within the year before and the year after PTNS treatment. Financial data included the costs of physicians, nurses, and medications. We calculated the costs of visits for physicians and nurses by using plans of Medicare, a US national social insurance program.

Finally, we compared the episodes of urinary symptoms and numbers of follow-up visits and analyzed the difference in the cost of the medications and visits before and after PTNS treatment.

### Statistical analysis

Descriptive statistics for continuous variables are presented as mean ± standard deviation, and categorical variables are presented as numbers and percentages. We performed statistical analysis by applying the Wilcoxon signed-rank test and paired samples t-test for nonparametric and parametric sample comparison for treatments before and after PTNS, respectively. The results were analyzed with a 95% confidence interval and p<0.05 was regarded as statistically significant. For the statistical analysis, we used SPSS version 16.0 (SPSS Inc., Chicago, IL, USA).

## Results

**Figure 1:**
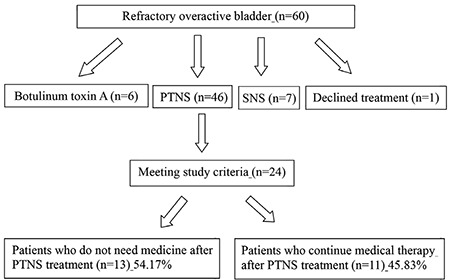
Flow of patients through the trial PTNS: Percutaneous tibial nerve stimulation; SNS: Sacral nerve stimulation

A total of 60 patients with a diagnosis of refractory OAB according to the International Continence Society were reviewed. Of the 60 patients, 6 were treated with intradetrusor botulinum toxin A (Botox®) injection, 7 were treated with SNS, 46 underwent at least 1 PTNS session, and 1 patient declined the third-line treatment. Of the 46 patients, 24 met the inclusion criteria of the study (Figure 1).

The mean age of the patients was 70.25±11.14 years. The mean BMI was 30.46±6.86 kg/m2. After initial PTNS therapy, 54.17% of the patients required no pharmacotherapy. The number of urgency episodes decreased from 3.12 to 1.79 (p=0.001), frequency episodes from 7.29 to 5.58 (p=0.002), leakage episodes from 2.62 to 1.33 (p=0.002), and nocturia episodes from 3.33 to 2.17 (p=0.01) (Table 1). The mean number of follow-up visits decreased from 1.34±0.78 to 0.40±0.63 after PTNS (p=0.001). After PTNS treatment, quarterly medication, nurse, and physician costs significantly decreased (Table 2). The quarterly total provider cost was calculated by adding the cost of follow-up visits to the cost of PTNS maintenance visits. The quarterly total provider cost was similar before and after PTNS treatment (p=0.143). However, the total provider cost for the last quarter decreased from $169.04±103.69 to $55.14±94.62 (p=0.005).

## Discussion

The results of our study showed that urinary symptoms were improved and the costs of medications and physician and nurse visits were reduced significantly in patients with refractory OAB who were treated with PTNS.

OAB syndrome is composed of symptoms that have significant effects on quality of life. Although urinary frequency, urgency, and nocturia are common symptoms, the main symptom is urgency (8). Patients are in fear of needing to urinate or urinary incontinence. From the patient’s perspective, OAB is a chronic and feared condition due to these symptoms; they feel boxed in and embarrassed (9).

PTNS is a third-line treatment for patients with refractory OAB. This therapy is effective in the alleviation of lower urinary tract symptoms such as incontinence, urgency, and frequency by electrical stimulation to afferent fibers of the posterior tibial nerve to the sacral center of micturition (6). Congregado Ruiz et al. (10) showed that leakage and daytime and nighttime frequency episodes were significantly improved after PTNS treatment (10). van Balken et al. (11) also found a positive response with PTNS therapy in terms of leakage episodes, voiding frequency, and nocturia. The Study of Urgent PC vs Sham Effectiveness in Treatment of Overactive Bladder Symptoms (SUmiT) trial, which was a double-blind, sham-controlled randomized trial, demonstrated the superiority of PTNS compared with sham groups. It reported moderate or marked improvement in bladder symptoms (54.5% in the PTNS-treated patients and 20.9% in the sham group). The study showed that PTNS reduced the number of voids, urgency, and incontinence episodes per day (12). We also found that number of urgency, frequency, leakage, and nocturia episodes significantly improved after PTNS treatment, similar to the literature.

The estimated health care cost of OAB in the USA is more than $65 billion per year (13). The total medical cost of patients with OAB receiving antimuscarinic therapy is estimated to be approximately $2000 per year (14). In our study, quarterly drug costs fell by almost half (from $656.36±292.45 to $375.51±331.79) after PTNS treatment. We also found a significant reduction in physician and nurse visit costs as a result of the decrease in the number of follow-up visits. Although quarterly total provider costs, including cost of follow-up visits and PTNS maintenance visits, were statistically similar before and after PTNS (p=0.143), the last quarter cost was significantly decreased from $169.04±103.69 to $55.14±94.62 (p=0.005). We concluded that after the initial 12 weeks of PTNS treatment, diminishing requirements for ongoing therapy towards the end of treatment led to the decrease in the last quarter cost. To the best of knowledge, there are no pure cost analysis studies of PTNS in patients with refractory OAB syndrome in the literature. Staskin et al. (15) reviewed the costs and effectiveness of PTNS in the current treatment of OAB. The authors recommended PTNS as an efficacious, less costly, and less invasive therapy compared with SNS for patients with OAB and refractory to antimuscarinic therapy. Although our study is also not an exact cost analysis of PTNS, we noted a reduction in the costs of medications and physician and nurse visits after PTNS treatment.

Our study has some limitations due to its retrospective design. However, the recall bias risk is low because all retrospective data related to the patient treatments and costs were objective data obtained from electronic patient files with no need for recall. Secondly, the size of the study is too small and follow-up times of the patients are relatively short for the assessment of long-term outcomes of PTNS treatment in patients with refractory OAB. On the other hand, the strength of our study is that it adds new results to the current literature on the changes in urinary symptoms and various costs of PTNS treatment in these patients.

In conclusion, PTNS therapy improves urgency, frequency, nocturia, and leakage episodes in patients with refractory OAB in the short term. This treatment also reduces the costs of medications and physician and nurse visits in the follow-up of these patients.

## Figures and Tables

**Table 1:**
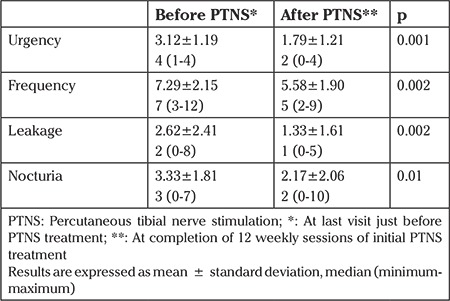
Changes in urinary symptom episodes of patients with refractory overactive bladder

**Table 2:**
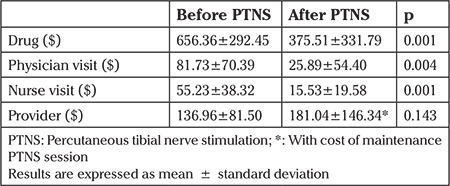
Comparison of quarterly costs before and after percutaneous tibial nerve stimulation treatment
